# A Rare Case of Early Gastric Mixed Neuroendocrine-Non-Neuroendocrine Neoplasm

**DOI:** 10.5152/tjg.2025.24574

**Published:** 2025-03-25

**Authors:** Shouxin Yin, Shuai Han, Shuangshuang Ren

**Affiliations:** 1 Department of Gastroenterology, People’s Hospital Affiliated to Shandong First Medical University, Shandong, China; 2 Department of Pathology, People’s Hospital Affiliated to Shandong First Medical University, Shandong, China

Dear Editor,

The mixed neuroendocrine-non-neuroendocrine neoplasms (MiNENs) are rare neoplasms that represent less than 1% of gastric cancer patients.^1^^-^^3^ They exhibit aggressive oncological behavior, a high frequency of vascular invasion and lymph node dissemination, leading to a poor prognosis.^3^ The early detection and diagnosis of MiNENs are usually difficult. A rare case of early gastric MiNEN is presented herein.

A 68-year-old man was hospitalized due to abdominal discomfort. Written informed consent was obtained from the patient who agreed to take part in the study. An esophagogastroduodenoscopy revealed a depressed lesion (Paris classification type 0-IIc) measuring 1.0 × 0.5 cm in size on the gastric angle (Figure 1A). Magnifying endoscopy revealed that the lesion had mostly clear boundaries, with an irregular microsurface pattern and microvascular pattern. However, some areas showed unclear boundaries and obscure microsurface pattern, along with dilated vessels (Figure 1B). Biopsy indicated high-grade intraepithelial neoplasia. The lesion was submitted to en bloc resection by endoscopic submucosal dissection. Histological examination revealed a MiNEN (Figure 2A). Within the mucosa, well-differentiated tubular adenocarcinoma was observed (Figure 2B). In the muscularis mucosa and submucosa, nest-like, sieve-like, and glandular tumor structures were present (Figure 2C), which were positive for neuroendocrine markers Syn, INSM1, and ATRX. The neuroendocrine component was classified as G3, with a Ki-67 proliferation index of 30% and wild-type P53. Examination of the NET component revealed a positive vertical margin. A distal gastrectomy with lymphadenectomy was performed, revealing no residual tumor or lymph node metastasis. Postoperative imaging studies have shown no evidence of recurrence at a follow-up time of 4 years.

Mixed neuroendocrine-non-neuroendocrine neoplasms should contain both neuroendocrine and non-neuroendocrine components, with each component comprising not less than 30%. They exhibit aggressive behavior, a high frequency of vascular invasion and lymph node metastasis. Compared with patients with pure gastric adenocarcinoma or neuroendocrine carcinoma, patients with MiNEN may have a poorer prognosis. Therefore, early diagnosis and treatment are crucial for improving prognosis. Early gastric MiNEN is defined as a tumor with an infiltration depth not exceeding the submucosal layer. Including this case, no more than 20 cases of early gastric MiNEN have been reported.^4^ A preoperative diagnosis of early gastric MiNEN is extremely challenging because the neuroendocrine component is always localized in the deepest part of the tumor and difficult to detect by white light endoscopy or even biopsy. White light endoscopy in early gastric MiNEN typically shows a submucosal, tumor-like elevation, while magnifying endoscopy reveals loss of microsurface pattern and irregular vessel dilation, which can also be observed in poorly differentiated adenocarcinomas.^4^^,^^5^ In addition, the neuroendocrine component may be misdiagnosed as poorly differentiated adenocarcinoma due to the similarity and the insufficient collection of biopsy samples. Therefore, if signs of gastric poorly differentiated or undifferentiated carcinoma are found, the possibility of MiNEN should be considered and immunostaining should be performed. In this case, the obscure local boundaries and microsurface pattern, combined with tortuous and dilated microvessels, suggest the possible presence of poorly differentiated adenocarcinoma or a neuroendocrine component. Preoperative biopsies and immunohistochemistry should be increased in this area to verify a neuroendocrine component. At present, considering the high incidence of lympho-vascular invasion even at an early stage, surgical resection rather than endoscopic submucosal dissection may provide better outcomes.^4^ However, we firmly believe that there is a curable stage for gastric MiNENs by endoscopic means, much like other gastrointestinal neoplasms. More studies are urgently needed to explore better diagnosis and treatment options for patients with gastric MiNEN.

A rare case of early gastric MiNEN is presented. Despite its difficulty, a precise preoperative diagnosis should be aimed for as possible, as this can lead to the best treatment for gastric MiNEN patients.

## Figures and Tables

**Figure 1.**
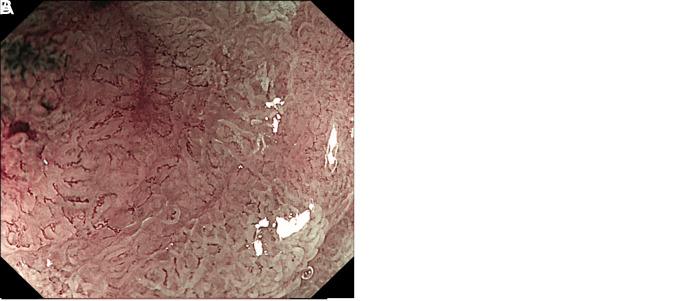
A. An esophagogastroduodenoscopy revealed a depressed lesion (Paris classification type 0-IIc) measuring 1.0 × 0.5 cm in size on the gastric angle. Biopsy indicated high-grade intraepithelial neoplasia. B. Some areas of the lesion showed unclear boundaries and obscure microsurface pattern, along with dilated vessels.

**Figure 2.**
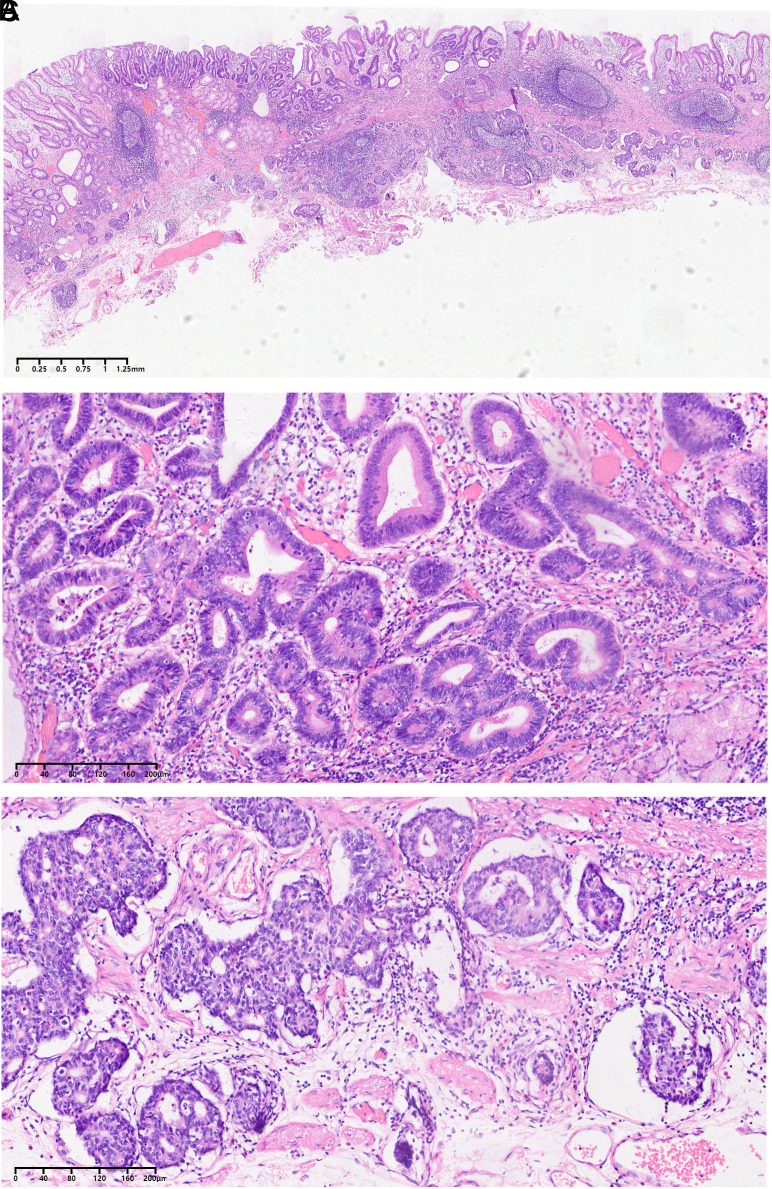
A. Histological examination of the ESD specimen revealed a mixed neuroendocrine-non-neuroendocrine neoplasm. B. Within the mucosa, well-differentiated tubular adenocarcinoma was observed. C. In the muscularis mucosa and submucosa, nest-like, sieve-like, and glandular tumor structures were present, which were diagnosed G3.

## Data Availability

The data that support the findings of this study are available on request from the corresponding author.
